# A molecular phylogenetic framework for *Bacillus subtilis* using genome sequences and its application to *Bacillus subtilis* subspecies *stecoris* strain D7XPN1, an isolate from a commercial food-waste degrading bioreactor

**DOI:** 10.1007/s13205-016-0408-8

**Published:** 2016-03-28

**Authors:** Joseph Adelskov, Bharat K. C. Patel

**Affiliations:** Microbial Gene Research and Resources Facility, School of Natural Sciences, Griffith University, Brisbane, QLD 4111 Australia

**Keywords:** Moderate thermophile, *Bacillus subtilis* subspecies, Phylogenomics, *Bacillus subtilis* subspecies *stecoris*

## Abstract

A thermophilic, heterotrophic and facultatively anaerobic bacterium designated strain D7XPN1 was isolated from Baku BakuKing™, a commercial food-waste degrading bioreactor (composter). The strain grew optimally at 45 °C (growth range between 24 and 50 °C) and pH 7 (growth pH range between pH 5 and 9) in Luria Broth supplemented with 0.3 % glucose. Strain D7XPN1 tolerated up to 7 % NaCl and showed amylolytic and xylanolytic activities. 16S rRNA gene analysis placed strain D7XPN1 in the cluster represented by *Bacillus subtilis* and the genome analysis of the 4.1 Mb genome sequence determined using RAST (Rapid Annotation using Subsystem Technology) indicated a total of 5116 genomic features were present of which 2320 features could be grouped into several subsystem categories. Of these, 615 features were related to carbohydrate metabolism which included a range of enzymes with potential in the biodegradation of food wastes, a property consistent with the ecological habitat of the isolate. ANIb (Average Nucleotide Identity based on BLAST) analysis with 49 *Bacillus subtilis* genomes indicated that it was distantly related to the three currently taxonomically validated *B. subtilis* subspecies namely *B. subtilis* subsp. *subtilis* (95.6 %), *B. subtilis* subsp. *spizizenii* (93 %) and *B. subtilis* subsp. *inaquosorum* (92 %) and based on our current knowledge warranted that it be included as a separate cluster together with strain JS which it was closely related (98.69 %). The close relationship of strains D7XPN1 and JS is also supported from our results from electronic DNA–DNA Hybridization (e-DDH) studies. Furthermore, our additional in-depth phylogenomic analyses using three different datasets unequivocally supported the creation of a fourth *B. subtilis* subspecies to include strains D7XPN1 and JS for which we propose strain D7XPN1^T^ (=KCTC 33554^T^, JCM 30051^T^) as the type strain, and designate it as *B. subtilis* subsp. *stecoris*.

## Introduction

The members of the genus *Bacillus* comprise the low G+C containing Gram-positive bacteria and belong to the family *Bacillaceae*, order *Bacillales*, class *Bacilli*, phylum *Firmicutes,* domain *Bacteria*. They grow in a diverse range of habitats and include species that grow at extreme temperatures, salinities and pH. The phenotypic traits and physiologies of members of the genus *Bacillus* are as dynamic and wide ranging as their habitats and include species that grow as heterotrophs, autotrophs, in the presence or absence of oxygen and yet others grow in the presence of alternate electron acceptors such as iron, arsenate (Kanso et al. 2002). The extreme metabolic diversity, their non-pathogenic nature and the ease of isolating, handling and maintainance has opened them to biotechnological exploitation and in particular for use in agriculture, e.g. crop protection (Gao et al. 2015; Hussein et al. 2015; Ji et al. 2008). 16S rRNA-based taxonomy is routinely used to build taxonomic relationship amongst members of the domain *Bacteria* but it is well established that this technique fails to resolve closely related *Bacillus* species in which evolutionary divergence is limited. Given the lack of discriminatory power of 16S rRNA genes, a polyphasic taxonomic approach which combines 16S rRNA sequence analysis with phenotypic traits and DNA–DNA homology has been recommended to improve resolution. Examples of such an approach include the members of the *Bacillus cereus* sensu lato group which currently comprises of 6 species (*B. cereus*, *B. anthracis*, *B. thuringiensis*, *B. mycoides* and *B. psedomycoides* and *B. weihenstephanensis* (Zwick et al. 2012) and the *B. subtilis* group which comprises of 3 species *B. subtilis*, *B.* *vallismortis and B. mojavensis* (Roberts et al. 1994, 1996). However, the topology of polyphasic trees is not always robust and can differ from the 16S rRNA based trees and therefore delineating species and strain boundaries using a polyphasic approach can be confusing. With the advent of the high throughput cost effective Next Generation Sequencing (NGS) technologies, estimation of overall similarity of microbial genomes by Genome-to-Genome Distance Comparison (GGDC), Average Nucleotide Identity (ANI) and genome phylogeny are being considered not only for delineating closely related species but also for taxonomic assignment of new isolates (Maughan and Van der Auwera 2011; Yi et al. 2014). Here we describe the construction of a molecular phylogenetic framework for *Bacillus subtilis* using genome sequences and its application to strain D7XPN1, an isolate from Baku Baku King™, a commercial food-waste degrading bioreactor (Adelskov 2013; Adelskov and Patel 2014).

## Materials and methods

### Bioreactor operation and sample collection

Biodegradation of food-wastes was carried out using Baku Baku King™ (model: M.I.G.0100), a food waste bioreactor. The biodegradation process was started in the bioreactor (day 0) by mixing 50 kg of municipal food waste sourced from a local hotel (Novotel Hotel, Gold Coast) with 200 kg of methyl bromide treated Japanese Larch wood chips and a microbial starter seed culture. The starter microbial seed culture was prepared by mixing 10 kg of leaf litter (collected from the Forest reserve located at Griffith University Nathan campus, Brisbane, Australia), with nutrients (sugar, honey, milk, meat, fish) followed by incubation for one week at room temperature (25–30 °C) before inoculation into the bioreactor. The bioreactor was subsequently fed 50 kg of municipal food waste per day sourced from the same hotel over a 49 day period. Samples (approximately 500 g) were collected from the bioreactor every 7–10 days of its normal operation cycle of 49 days including day 0 (the day of inoculation of the bioreactor with a starter seed culture). In total, seven samples were collected (days 0, 1, 7, 17, 21, 29 and 49). The temperature and aeration in the bioreactor was controlled by the inbuilt bioreactor’s electronic system.

### Enrichment, isolation and phylogeny

1 g of bioreactor samples was resuspended in 10 ml sterile dH_2_O the solution shaken for 2 min and the debris allowed to settled for 5 min. 200 µl of settled suspension was spread on dTSA agar medium [dTSA consisted of 0.1 % (w/v) Tryptic Soy Broth, 1.5 % (w/v) bacteriological agar, pH 7.2]. The temperature of the bioreactor during most of the operation period was between 40 and 45 °C and hence the plates were incubated at 45 °C until colonies developed. Single well-isolated colonies that appeared morphologically distinct were picked and resuspended in sterile dH_2_O and a loopful streaked onto dTSA agar plates and incubated at 45 °C until colonies developed. This procedure was repeated several times before the isolates were considered to be pure. 28 pure cultures (Table 1) were obtained using this process and were stored at 4 °C and −20 °C. All isolates were routinely cultured using 0.1 % (w/v) (dilute) Tryptic Soy Broth (TSB), pH 7.2.

**Table 1:** List of strains isolated from the waste-food degrading bioreactor, Baku Baku™

Day	Isolate	Colony morphology^a^	Enzyme activity^b^		Closest phylogenetic member^c^
			Xylanase	Amylase	
0	BXP1	Irregular, white, 2–3 mm	–	+	*Bacillus subtilis* str. 168
0	BXP7	Slighly irregular, mucoid, white, 2–3 mm	–	+	*Bacillus vallismortis* str. DSM 11031
1	BXP11	Circular, white, 1 mm	+	+	*Paenibacillus cookii* str. LMG 18419
1	BXP12	Circular, irregular margin, 1 mm	–	+	*Bacillus subtilis* str. DSM 10
1	BXP13	Circular, white, 1–2 mm	–	+	*Bacillus subtilis* str. 168
7	D7XPN1	Circular, irregular margin, white, 1 mm	+	+	*Bacillus subtilis* str. 168
16	D16IS1	Irregular, filamentous margin, white, 5–6 mm	–	+	*Bacillus aerius* str. 24 K
16	D16IS2	Circular, mucoid, white, 1.5–2 mm	–	+	*Bacillus subtilis* str. DSM 10
16	D16IS3	Rhizoid, white, 4–6 mm	–	+	*Bacillus licheniformis* str. ATCC 14580
16	D16IS5	Filamentous, translucent	–	–	*Kurthia gibsonii* str. NBRC 15534
16	P1XP2	Circular, mucoid, white, 0.7 mm	+	+	*Paenibacillus cookii* str. LMG 18419
16	P1XP3	Irregular, white, 1–3 mm	–	+	*Bacillus subtilis* str. DSM 10
16	P1XP5	Circular,mucoid, white, 0.8 mm	+	+	*Paenibacillus cookii* str. LMG 18419
21	D21IS1	Circular, mucoid, white, 1–1.2 mm	–	+	*Paenibacillus ehimensis* str. KCTC 3748
21	D21IS2	Irregular, slighly opague white, 2 mm	–	+	*Bacillus licheniformis* str. ATCC 14580
21	D21IS3	Rhizoid, white, 3 mm	–	+	*Bacillus licheniformis* str. ATCC 14580
21	D21IS4	Circular, irregular margin, white 3–4 mm	–	+	*Bacillus subtilis* str. DSM 10
29	D29IS4	Circular, mucoid, white, 1 mm	+	+	*Paenibacillus cookii* str. LMG 18419
29	D29IS5	Circular, irregular margin, white, 2–3 mm	–	+	*Bacillus subtilis* str. DSM 10
29	D29IS6	Irregular, slightly opaque, white, 4–5 mm	+	+	*Bacillus licheniformis* str. ATCC 14580
35	D35IS1	Irregular, filamentous margin, white, 6 mm	–	+	*Bacillus aerius* str. 24 K
35	D35IS3	Irregular, white, 1.2 mm	–	–	*Brevibacillus agri* str. DSM 6348
35	D35IS5	Filamentous, translucent, spreading	–	–	*Bacillus badius* str. 110
42	D42IS3	Circular, mucoid, white, 1.2 mm	+	+	*Paenibacillus cookii* str. LMG 18419
49	D49IS3	Irregular, white, 2–4 mm	–	+	*Bacillus subtilis* str. DSM 10
49	D49IS4	Circular, filamentous margin, white, 3–4 mm	–	+	*Bacillus licheniformis* str. ATCC 14580
49	D49IS5	Circular, slightly translucent, mucoid, 0.5 mm	–	–	*Aneurinibacillus migulanus* str. B0270

^a^The strains were isolated on 0.1 % (dilute) Tryptic Soy Agar (dTSA) medium and colony morphology noted after 48 h incubation at 45 °C

^b^Xylanase and amylase activities were determined as described in “Materials methods” Section

^c^The closest phylogenetic match was determined by BLAST analysis of the 16S rRNA sequences of the isolates against the NCBI 16S ribosomal RNA (*Bacteria* and *Archaea*) database

The 28 isolates were cultured in TSB (pH 7.0) at 45 °C for 18 h, the cells centrifuged and the DNA from the pelleted cells purified using a modification of Marmur’s method as described by Ogg and Patel (2009). In brief, bacterial cells were resuspended in a buffered solution (50 mM Tris, 10 mM EDTA, pH 7.8) and treated with 0.8 mg/ml lysozyme, 0.3 mg/ml Achromopeptidase and 0.1 mg/ml RNAse A and subsequently lysed by adding 0.12 mg/ml Proteinase K and 6 mg/ml sodium dodecyl sulphate (SDS) to the suspension. The DNA from the lysate was purified using phenol:chloroform extraction. Purified DNA quality was assessed by agarose gel electrophoresis and DNA concentration determined fluorometrically using a Qubit™ dsDNA HS assay kit as described by the manufacturer (Life Technologies, USA). The 16S rRNA gene was amplified from the DNA of the isolate by PCR using the universal forward primer Fd1 (AGAGTTTGATCCTGGCTCAG) and reverse primer Rd1 (AAGGAGGTGATCCAGCC) that bind to the 8–27 and 1512–1493 base pair positions of *E. coli* numbering scheme according to Winkler and Woese (1991). Reactions of 50 µl volume consisted of: 0.2 mMdNTP, 2 mM MgCl_2_, 1 mM Fd1, 1 mM Rd1, 0.5–5 ng of DNA template, 2.5 U of Taq polymerase (Mango Taq) and provided reaction buffer. PCR proceeded using a Corbett Research FTS-1 Thermal sequencer with the following cycle program: cycle 1; 2 min 95 °C; 1 min 50 °C; 2 min 70 °C, cycle 2–32; 55 s 94 °C, 1 min 50 °C, 2 min 72 °C. The reaction amplicon was purified by either SureClean™ or Gel extraction (QIAGEN) following manufacturer’s instructions. The purified amplicons were sequenced on an ABI 3730xl 96-capillary sequencer using Fd1 and Rd1 primers at AGRF (Australian Genetics Research Facility). 16S rRNA gene sequence manipulation and phylogenetic analysis was performed as described previously (Redburn and Patel 1994).

All isolates were screened for the presence of amylase activity by inoculating a loopful of culture onto dTSA agar medium supplemented with 1 % soluble potato starch (Chem Supply, Australia) and incubation at 45 °C until colonies developed. The plates were flooded with Gram’s Iodine solution and a positive reaction for amylase production recorded for isolates when there was a zone of clearance around colonies against a red–purple background. Isolates were screened for xylanase activity by streaking X-xyl Agar plates followed by incubation at 45 °C. X-xyl Agar plates contained (g^−L^ distilled water): Tryptic Soy Broth (Oxoid, USA) 1 g, xylan from birchwood (Sigma, USA) 3 g, Bacteriological Agar (Oxoid, USA) 15 g, X-β-D-xyloside (Gold Biotech, USA) 0.2 g, pH 7.2 and incubated at 45 °C. Xylanase production was recorded as positive when colonies showed a blue color.

One of the isolates designated D7XPN1, which produced a xylanase and amylase, was selected for further studies and is described in more detail in this paper.

### Characterization of strain D7XPN1

Temperature, pH and salinity growth studies were conducted in 18 mm glass culture tubes containing 15 ml of modified Luria Bertoni Broth (mLBB). mLBB contained per litre Luria Bertoni Broth (Oxoid, USA) 25 g, d-Glucose anhydrous (Lab Supply, Australia) 3 g. For temperature studies, mLLB was inoculated with 0.2 ml of an overnight culture and incubated in water baths maintained at 37, 45, 50, 60, 70 °C and incubated for 48 h. For pH studies, the pH of mLBB was changed to the desired pH (pH range of 4.0–10) by addition of 1 M HCl or 1 M NaOH, inoculated with 0.2 ml of an overnight culture and incubated in water baths maintained at 45 °C. For salinity studies, appropriate amounts of NaCl was weighed and added to mLBB medium to achieve the desired concentration of salinity (3–7 %), inoculated with 0.2 ml of an overnight culture and incubated in a water bath maintained at 45 °C for 48 h. Following incubation, growth was determined by inserting the glass culture tubes directly into a modified cuvette holder of a Novaspec LKB spectrophotometer and the absorbance measured at 600 nm. Anaerobic growth was tested in Trypticase, Yeast Extract, Glucose (TYEG) medium as described previously (Ogg and Patel 2009).

### Dataset and genome sequencing

Unless indicated otherwise, all computational analysis was performed using a 16 CPU Dell workstation with 64 gigabytes RAM and an Intel^®^ Xeon(R) CPU X5570 @ 2.93 GHz × 8 chipset running Ubuntu 12.04 and the Australian Government Information Technology Infrastructure Facilities accessed under the National eResearch Collaboration Tools and Resources (NeCTAR) program.

Complete and draft Whole Genome Sequences (WGS) for all strains that were identified in GenBank microbial genome database as *Bacillus subtilis*, *B. amyloliquefaciens* and *B. atrophaeus* were downloaded from the NCBI ftp server ftp.ncbi.nlm.nih.gov/genomes/genbank/bacteria (release 204) (Table 2). Unless indicated otherwise, sequence contigs from all GenBank files were extracted and converted to fasta format (Vesth et al. 2013) and any plasmid sequences accompanying the genome data (*B. subtilis* subsp. *natto* str. BEST195, B. *subtilis* subsp. str. NCIB 3610 and B. *subtilis* subsp. *subtilis* str. B7-s) were removed before use in comparative genomic studies.

**Table 2:** *Bacillus subtilis* strains used in determining the Overall Genome Relatedness Indices (OGRI) using Genome-to-Genome Distance Calculations (GGDC) and Average Nucleotide Identity by Blast (ANIb) and phylogenomics

No.	*B. subtilis* strain	Accession Number	Sample source	Total nucleotide (bp)	No. of Contigs	Largest Contig size (%)	N25	N50	N75	G+C mol %
Cluster 1. *Bacillus subtilis* subsp. *subtilis*										
1	*Bacillus* *subtilis* subsp *subtilis* str. JH642 substr. AG174	CP007800	Not known	4,188,369	1	100	4,188,369	4,188,369	4,188,369	43.00
2	*Bacillus* *subtilis* subsp. *subtilis* str. AG1839	CP008698	Not known	4,193,640	1	100	4,193,640	4,193,640	4,193,640	43.48
3	*Bacillus* *subtilis* subsp. *subtilis* str. JH642*	CM000489	Laboratory strain	4,187,615	1	100	4,187,615	4,187,615	4,187,615	43.49
4	*Bacillus subtilis* str. PY79	CP006881	Laboratory strain	4,033,459	1	100	4,033,459	4,033,459	4,033,459	43.83
5	*Bacillus subtilis* subsp. *subtilis* str. QB928*	CP003783	Laboratory strain	4,146,839	1	100	4,146,839	4,146,839	4,146,839	43.61
6	*Bacillus subtilis* str. E72	JNCN01000000	Laboratory strain	4,168,037	35	28.242	1,177,131	1,017,254	487,713	43.39
7	*Bacillus subtilis* subsp. *subtilis* str. 168*	AL009126	Not known	4,215,606	1	100	4,215,606	4,215,606	4,215,606	43.51
8	*Bacillus subtilis* subsp. *subtilis* str. SMY*	CM000490	Not known	4,214,643	1	100	4,214,643	4,214,643	4,214,643	43.51
9	*Bacillus subtilis* subsp. *subtilis* str. NCIB_3610 (T)*	CM000488	Not known	4,292,969	80	98.174	4,214,598	4,214,598	4,214,598	43.51
10	*Bacillus subtilis* subsp. *subtilis* str. NDmed	JPVW01000000	Endoscope disinfector, UK	4,059,983	10	29.391	1,193,268	939,235	438,498	43.69
11	*Bacillus subtilis* subsp. *subtilis* str. NDfood	JPVX01000000	Dairy product, France	4,060,577	12	26.097	1,059,688	556,411	438,563	43.69
12	*Bacillus subtilis* subsp. *subtilis* str BEST7003	CP007800	Not Known	4,043,042	1	100	4,043,042	4,043,042	4,043,042	
13	*Bacillus subtilis* str. MB73-2	AOTY01000000	Meadow soil, Poland	4,170,657	35	35.757	1,491,311	1,017,190	926,340	43.43
14	*Bacillus subtilis* subsp. *subtilis* str. 6051-HGW	CP003329	Not known	4,215,610	1	100	4,215,610	4,215,610	4,215,610	43.51
15	*Bacillus subtilis* str. PS216	AQGR01000000	Sandy soil from Sava river bank, Slovenia	4,307,186	146	21.585	298,458	177,899	105,241	43.77
16	*Bacillus subtilis* subsp. *subtilis* str. AUSI98*	JH600074	Soil, Austria	4,353,151	123	5.516	181,423	128,442	59,891	43.48
17	*Bacillus subtilis* str. QH-1	AZQS01000000	Soil, China	4,034,036	11	25.876	1,043,843	1,043,657	1,029,685	43.71
18	*Bacillus subtilis* subsp. *subtilis* str. SC-8*	AGFW01000000	Korean traditional fermented-soybean, Korea	4,138,818	17	27.059	1,119,942	8,37,849	252,500	43.46
19	*Bacillus subtilis* str. GXA-28	JPNZ01000000	Marine sand, China	4,261,419	13	27.141	1,156,606	110,0410	1,052,768	43.59
20	*Bacillus subtilis* subsp. *subtilis* str. OH	CP007409	Wheat anther, USA	4,039,155	1	100	4,039,155	4,039,155	4,039,155	43.85
21	*Bacillus subtilis* subsp. *subtilis* str. BSn5*	CP002468	*Amorphophallus konjac* calli tissue culture	4,093,599	1	100	4,093,599	4,093,599	4,093,599	43.85
22	*Bacillus subtilis* str. KATMIRA1933	JMEF01000000	Yogurt- cultured beverage, USA	4,263,792	125	5.031	139,190	90,789	43,123	43.38
23	*Bacillus subtilis* subsp. *subtilis* str. BSP1	CP003695	Not known	4,043,754	1	100	4,043,754	4,043,754	4,043,754	43.87
24	*Bacillus subtilis* str. PTS-394	AWXG01000000	Tomato rhizosphere soil, China	4,005,786	34	25.38	1,016,662	471,675	239,700	43.69
25	*Bacillus subtilis* subsp. *subtilis* str. BEST195*	AP011541	Natto production strain, Japan	4,111,218	2	99.858	4,105,380	4,105,380	4,105,380	43.50
26	*Bacillus subtilis* str. S1-4	ANIP01000000	Feathers from a poultry farm, China	4,454,050	104	6.288	152,724	88,085	54,963	43.08
27	*Bacillus subtilis* subsp. *subtilis* str. MP9	APMW01000000	*Macrotermes natalensis*, South Africa	3,952,500	323	2.905	35,773	23,097	12,323	43.78
28	*Bacillus subtilis* subsp. *subtilis* str. MP11	APMX01000000	*Macrotermes natalensis*, South Africa	3,930,802	647	1.58	18,223	11,158	6272	43.69
29	*Bacillus subtilis* str. E1	CAUC01000000	Identified as a plant growth promoting bacterium	4,106,253	16	28.556	1,172,592	987,088	332,060	43.55
30	*Bacillus subtilis* str. Hal1	AMCA01000000	River water, India	3,979,047	345	3	36,650	22,550	12,343	43.79
31	*Bacillus subtilis* str. XF-1	CP004019	Rhizosphere of chinese cabbage, *Brassica pekinensis* infected with *Plasmodiophora brassicae*	4,061,186	1	100	4,061,186	4,061,186	4,061,186	43.86
32	*Bacillus subtilis* subsp. *subtilis* str. BAB-1	CP004405	Cotton rhizosphere	4,021,944	1	100	4,021,944	4,021,944	4,021,944	43.89
33	*Bacillus subtilis* subsp. *subtilis* str. RO-NN-1*	CP002906	Soil	4,011,949	1	100	4,011,949	4,011,949	4,011,949	43.87
Cluster 2. *Bacillus subtilis* subsp. *stecoris*										
34	*Bacillus subtilis* str. D7XPN1 (This study)	JHCA00000000	Food waste bioreactor (Baku Baku), Australia	4,079,419	28	24.943	1,010,461	504,008	237,557	43.80
35	*Bacillus* sp. JS	CP003492	Pot soil of *Miscanthus*, Korea	4,120,406	1	100	4,120,406	4,120,406	4,120,406	43.93
Cluster 3. *Bacillus subtilis* subsp. *inaquosorum*										
36	*Bacillus subtilis* subsp. *inaquosorum* str. KCTC_13429 (T)*	AMXN01000000	South Korea	4,342,448	24	19.076	744,296	566,730	272,952	43.69
37	*Bacillus subtilis* str. gtP20b*	AEHM01000000	608-m deep sediment, Indian Ocean	4,205,767	88	13.052	284,523	221,307	70,214	43.99
Cluster 4. *Bacillus subtilis* subsp. spizizenii										
38	*Bacillus subtilis* subsp. *spizizenii* str. TU-B-10 (T)*	CP002905	Soil, Tunisia	4,207,222	1	100	4,207,222	4,207,222	4,207,222	43.82
39	*Bacillus subtilis* subsp. *spizizenii* str. DV1-B-1*	AFSG01000000	Soil, Death Valley National Monument USA	3,974,551	20	20.692	789,480	684,333	279,652	43.60
40	*Bacillus subtilis* str. BSC154	JPWY01000000	Biological Soil Crust, USA	4,028,151	19	18.739	660,959	634,763	223,155	43.72
41	*Bacillus subtilis* subsp. *spizizenii* str. W23*	CP002183	Laboratory strain	4,027,676	1	100	4,027,676	4,027,676	4,027,676	43.89
42	*Bacillus subtilis* subsp. *spizizenii* str. ATCC_6633*	ADGS01000000	Laboratory strain	3,978,576	37	8.234	269,373	170,545	122,278	43.82
43	*Bacillus subtilis* str. BST	KN049967	Laboratory strain	4,043,115	1	100	4,043,115	4,043,115	4,043,115	43.93
Isolates whose genomes show that they are not members of *Bacillus subtilis*										
44	*Bacillus subtilis* subsp. *niger* str. PCI**	KN049968	USAMRIID, USA	4,154,887	2	99.845	4,148,457	4,148,457	4,148,457	43.18
45	*Bacillus subtilis* str. GB03**	AYTJ01000000	Laboratory strain	3,849,547	37	20.369	485,347	387,471	227,849	46.55
46	*Bacillus subtilis* str. SPZ1**	AQGM01000000	Laboratory strain	4,134,697	79	14.214	453,273	201,909	119,673	46.00
47	*Bacillus subtilis* str. NKYL29**	JPYY01000000	Soil, China	3,951,956	22	23.903	940,749	592,536	270,804	46.33
48	*Bacillus* sp. EGD-AK10	AVPM01000000	Agricultural soil, India	6,565,134	1184	14.753	731,082	292,564	3046	55
49	*Bacillus subtilis* B7-s	AZNI00000000	China	5,313,924	82	9.097	ND	198,717	112,914	35.1

Genomes studied by Yi et al. (2014) which were identified as members of *B. subtilis* group are marked by a single asterisk (*)

The isolates which are listed in the NCBI database as members of *B. subtilis* but are clearly not as reported in our study here (Table 3), are marked by **

Type strains of *Bacillus subtilis* subspecies are identified with a (T)

**Table 3:** Genomes misclassified as *Bacillus subtilis* based on ANIb analysis

Strain	Mean ANI vs species		
	*B. subtilis*	*B. amyloliquefaciens*	*B. atrophaeus*
*Bacillus subtilis* str. GB03	0.79581	**0.97505**	0.79787
*Bacillus subtilis* str. SPZ1	0.7959	**0.97518**	0.79098
*Bacillus subtilis* str. NKYL29	0.7877	**0.97932**	0.7903
*Bacillus subtilis* subsp. *niger* str. PCI	0.8104	0.79287	**0.99912**

Numbers in bold signify the most likely associated species based on the mean of ANIb comparisons to geonomes of the three different species

Library construction and sequencing of the genome of strain D7XPN1 was performed at the Australian Genome Research Facility (AGRF) core facility on an Ion Torrent PGM sequencer using a 318 chip. The sequencing data was converted to FASTQ format and adapters were removed from individual reads. The quality of the sequencing data was assessed using PRINSEQ (http://prinseq.sourceforge.net/) (Schmieder and Edwards 2011). Genomic contigs were assembled from reads using the GS *de novo* Assembler (Newbler) software (http://454.com/products/analysis-software/index.asp). The assembled draft genome was annotated using Prokka, version 1.10 (Seemann 2014) and the RAST automated annotation pipeline server (http://rast.nmpdr.org/) (Aziz et al. 2008), which employs subsystems technology to identify genes related to different categories of cellular processes and metabolism (Overbeek et al. 2014). The whole-genome shotgun project of *Bacillus subtilis* strain D7XPN1 (= KCTC 33554, JCM 30051) has been deposited at DDBJ/EMBL/GenBank under the accession number JHCA00000000. The version described in this paper is version JHCA00000000.1.

### Estimation of overall genome relatedness indices (OGRI) for strain D7XPN1

OGRI methods depend on comparisons of whole genome rather than single genes or a set of genes and ANIb has been established as a method of choice. ANIb was performed with genome nucleotide sequences using the calculate_ani.py script (https://github.com/widdowquinn/scripts) that incorporates the ANI algorithm by Richter and Rossello-Mora (2009). In addition, intergenomic e-DDH distances were calculated using Genome-to-Genome Distance Calculator (GGDC) with the recommended formula as described by Meier-Kolthoff et al. (2013).

### Phylogenomics

The use of groups of orthologous proteins or a set of common conserved genes across genomes is required for phylogenomic analysis and several methods are available for such studies. In our study, we first used Prokka (version 1.10) (Seemann 2014) to identify open reading frames (ORF) of genome nucleotide sequences (*n* = 43), which were subsequently translated into putative protein sequences and annotated. We then created two protein datasets for phylogenomic studies. For the first, we used the hal pipeline with default settings to find and extract all protein orthologs (Robbertse et al. 2011) and for the second, we used CD-Hit (Huang et al. 2010) to select for a smaller but highly conserved set of orthologuous proteins from the annotations (>97 % AA identity). Additionally, we used the 6 conserved MLST gene dataset (glpF, pta, purH, pycA, rpoD and tpiA) typically used for typing strains of *Bacillus subtilis* for phylogenomic Multilocus Sequence Analysis (MLSA). The 6 genes were downloaded from pubMLST database and used in local BLAST queries against the *Bacillus subtilis* genome database of open reading frames (ORF) and the MLST genes retrieved. The datasets of the conserved protein sequences from hal and CD-Hit and the MLST nucleotide sequences were aligned separately using CLUSTALW (http://www.clustal.org/clustal2) (Larkin et al. 2007), concatenated into a single super-alignment and used to construct Maximum Likelihood trees using PhyML (Guindon et al. 2010).

## Results

### Strain isolation and phylogenetic identification

**Fig. 1:**
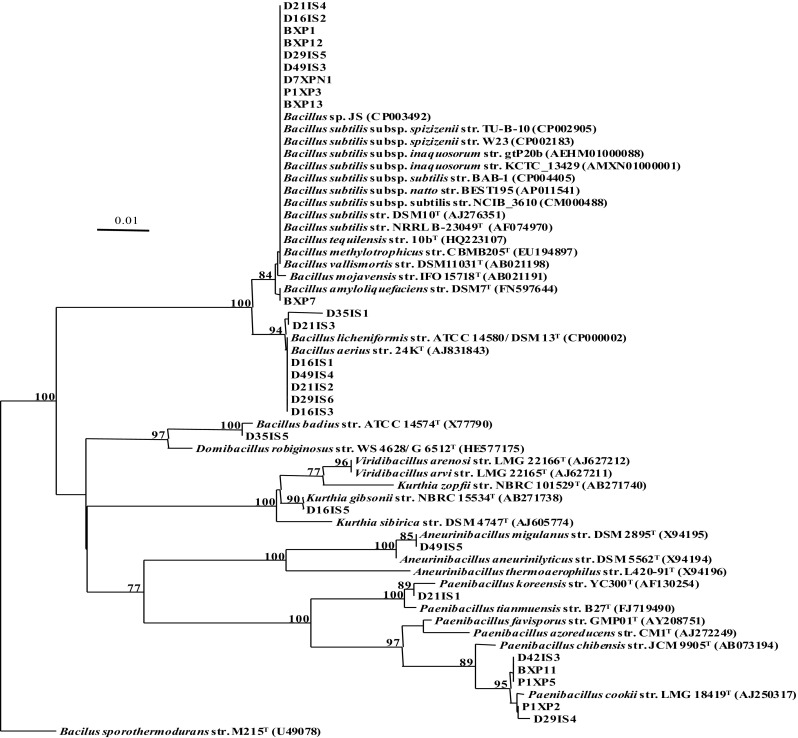
Phylogenetic distance tree constructed from partial 16S rRNA sequences (467 nucleotides) of bacterial strains isolated from samples retrieved from Baku Baku™ a food waste bioreactor. Distance estimation was obtained using the Jukes and Cantor model. Bootstrap percentages after 1000 replications are shown. Scale bar represents one nucleotide change in every 100 nucleotides. Refer to Table 2 for the type strains in this figure

28 strains were isolated from bioreactor samples collected over 49 days of operation. The strains were selected on the basis of differences in colony morphology, growth rates and enzyme screening (amylase and xylanase) (Table 1). 16S rRNA gene (sequence length between 560 and 1555 bp) analysis revealed that 27 of the 28 isolates were members of the genera *Bacillus, Paenibacillus, Kurthia* and *Aneurinibacillus*, phylum *Firmicutes*, domain *Bacteria* (Fig. 1) whereas the 28th isolate, identified using 18S rRNA gene sequence analysis (sequence length 1738 bp) analysis as *Ogataea polymorpha*, a yeast (data not shown). Further phylogenetic analysis showed that 9 of the isolates cultured from samples taken from the bioreactor on days 0, 1, 7, 17, 21, 29, and 49 of its operation cycle (Table 1), and which included strain D7XPN1, were closely related to *Bacillus subtilis* (99 % similarity) (Fig. 1).

### Phenotypic characterization of strain D7XPN1

The cells of strain D7XPN1 were short rods which stained Gram positive and produced cream coloured, opaque, raised, irregular-shaped colonies on dTSA medium. Strain D7XPN1 grew optimally at 45 °C (growth temperature between 24 and 50 °C) suggesting that it was a thermotolerant/moderate thermophile and pH 7 (pH growth range between pH 5 and pH 9). The strain tolerated up to 7 % NaCl (the highest tested) and grew anaerobically by fermentation in the absence of oxygen.

### Genome studies of strain D7XPN1

**Fig. 2:**
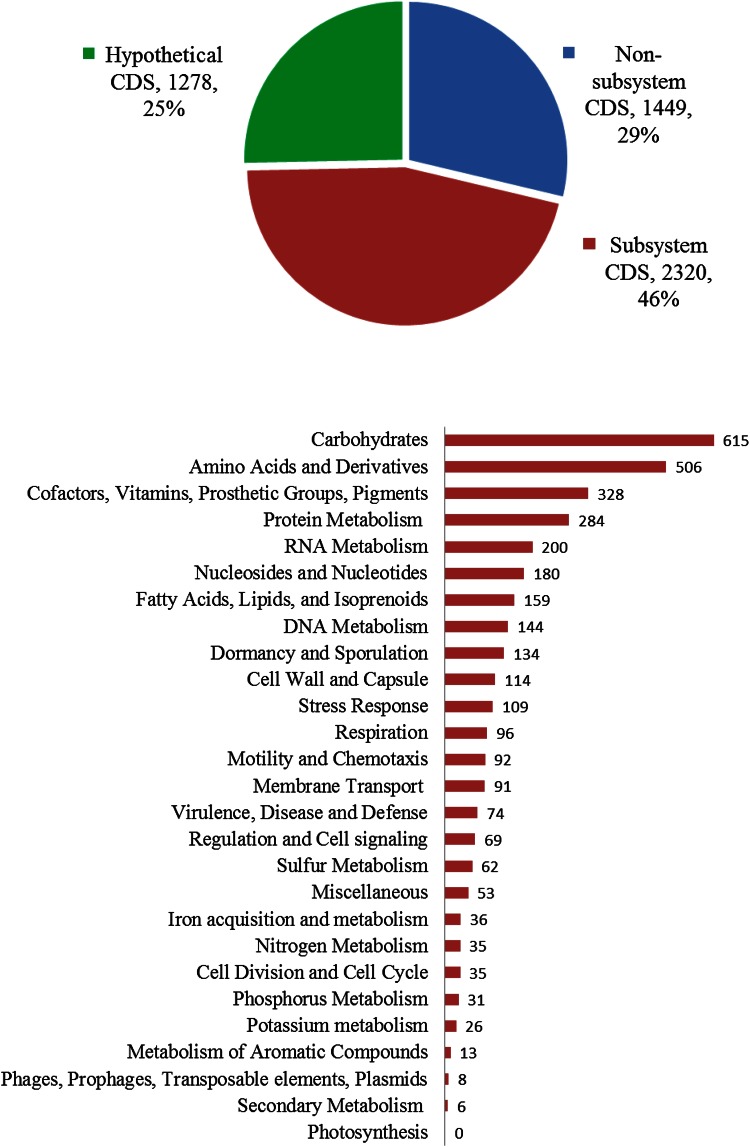
**a** RAST annotation gene functional categories of *Bacillus subtilis* str. D7XPN1. **a** The pie graph of the total numbers of CDS is divided into 3 categories (in %)—CDS annotations that are represented in the RAST subsystem, those that are not represented (non-subsystem), and those that annotations that are hypothetical. **b** The *bar graph* shows the total subsystem CDS annotations by major subsystem categories

A total 722,222 reads with a mean read length of 196.13 bp (total of 141,651,194 bp) were produced using IonTorrent™. The assembly of these reads with GS assembler (Newbler) produced 28 genomic contigs (average coverage of 40x) with contig sizes ranging from 1,017,528 to 510 bp in length and a N50 of 504,008 bp. RAST server identified a total of 5116 genomic features that included 69 RNA and 5047 protein coding sequences (Fig. 2). Of the 5047 total features, 2,320 were placed into functional subsystems. Two subsystems categories related to carbohydrates and amino acid and derivatives had the highest number of associated features with 615 and 506 coding features, respectively.

### Estimation of overall genome relatedness indices (OGRI)

**Fig. 3:**
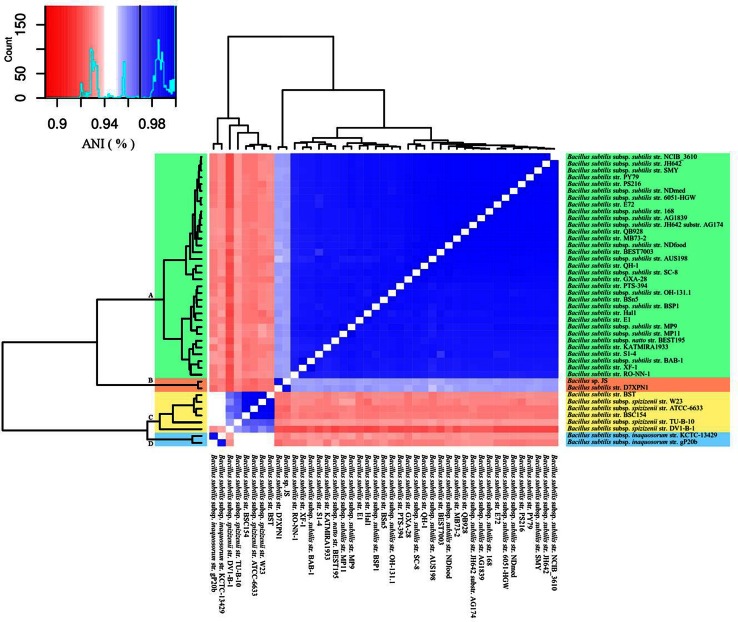
ANIb heatmap of *Bacillus subtilis* genome sequences (*n* = 43). Individual genome-to-genome ANIb values are represented in the central bi-color gradient heatmap, the color key shown on the top left had side including adivison line for the recommended subspecies cutoff value (97 %). The heatmap is accompanied by a hierarchical clustering dendrogram with for distinct clades (clusters): *B. subtilis* subsp. *subtilis* (A,* green*), *B. subtilis* subsp. *stecoris* (B,* pink*), *B. subtilis* subsp. *spizizenii* (C,* yellow*) and *B. subtilis* subsp. *inaquosorum* (D,* blue*). Refer to Table 2 for the type strains in this figure

49 strains which had a 16S rRNA gene sequence similarity of ≥97 % to *B. subtilis* strain 168, together with genomes of the members of *B. subtilis* sensu lato group, *B. atrophaeus* and strain D7XPN1 were initially used in the ANIb studies (Table 2). The study showed that 43 strains with an ANIb similarity values >92 % could be regarded as members of *B. subtilis* (Fig. 3) whereas the remaining 6 strains whose similarity values were <92 %, should be examined more closely. Closer examinations showed that *B. subtilis* strain GBO3, *B. subtilis* strain SPZ1 and *B. subtilis* str. NKYL29 shared a high ANIb similarity value (97 %) with *B. amyloliquefaciens* and *B. subtilis* subsp. *niger* str. PCI with *B. atrophaeus* (99.9 %) (Table 3). Additionally, review of the literature showed that the genome of *B. subtilis* strain BEST7613 was a chimeric construct of the genomes of *Synechocystis* strain PCC6803 and *B. subtilis* 168 (Watanabe et al. 2012) and the examination of the statistics of the genome of *B. subtilis* B7-S showed that it had a genome that was substantially different in size (5.3 Mb) and in G+C mol % content (35.1) to 43 members of the *B. subtilis* cluster. These 6 misclassified strains were therefore removed from further analysis.

**Fig. 4:**
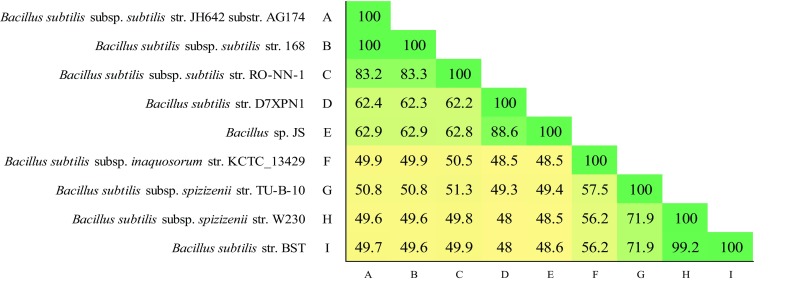
GGDC estimated DNA–DNA Hybridization (DDH, formula 2) of a selected set of *B. subtilis* genomic strains representing the three current sub-species and the proposed fourth sub-species

The remaining 43 strains could be further grouped into 4 clusters based on ANIb similarity values (Fig. 3). Cluster 1 constituted the largest group (33 strains) with a ANIb similarity value of >98 % amongst the members and is represented by the taxonomically validated *B. subtilis* subsp. *subtilis*. Of the 33 strains, 16 have already been identified as members of cluster 1. Cluster 2 consists of the two newly isolated strains JS (Song et al. 2012) and D7XPNI (Adelskov and Patel 2014), (Fig. 2) which have an ANIb similarity value of 95.6 % to cluster 1 and to each other by 98.8 %. These two strains have not been taxonomically validated previously and in this report we propose to describe these two strains as members of a new subspecies, *B. subtilis* subsp *stecori* of which strain D7XPN1^T^ is the type sub-species. Cluster 3 is composed of two strains of the taxonomically validated *B. subtilis* subsp. *inaquosorum* (Table 1) with an ANIb similarity value of 92 and 93 % to clusters 1 and 2, respectively and to each other by 98.6 %. The remaining 6 strains belong to a loose cluster represented by the taxonomically validated *B*. *subtilis* subsp. *spizizenii* which have an ANIb similarity value of between 92 and 94 % with members of clusters 1, 2 and 3. Of the 6 strains, 4 strains have been correctly identified as members of this cluster (Table 2). Of the 6 strains, 4 strains (*B. subtilis* str. BSC154, *B. subtilis* subsp. *spizizenii* str. W23, *B. subtilis* subsp. *spizizenii* str. ATCC 6633 and *B. subtilis* str. BST) group closely together (ANIb value of >99 %) whereas *B. subtilis* subsp. *subtilis* str. DV1-B-1 and *B. subtilis* subsp. *spizizenii* str. TU-B-10 are more distant (ANIb values of 95.6 and 92.2 %, respectively) to the 4 strains.

### DNA homology using genome-to-genome-distance-calculator (GGDC)

DNA–DNA hybridization (DDH) method is a gold standard that is used to differentiate species of the same genus when the 16S rRNA sequence similarity is >97 %. The widely accepted species boundary set by the DDH method is 70 %. The Genome-to-Genome-Distance-Calculator (GGDC) is an in silico alternate for the traditional experimental DDH method and is the second OGRI method used in our study. As all the *B. subtilis* strains (*n* = 43) have a 16S rRNA similarity value >97 %, we have calculated the GGDC similarity indices of representatives genomes from each of the 4 sub-species clusters. The results show that *B. subtilis* subsp. *stecori* strains D7XPN1 and JS of cluster 2 share a genome similarity of 88.6 % to each other and 62.2–62.9 % with strains of cluster 1 represented by *B. subtilis* subsp. *subtilis* and <51 % with strains from the clusters 3 and 4 represented by *B. subtilis* subsp. *spizizenii* and *B. subtilis* subsp. *inaquosorum* (Fig. 4).

### Phylogenomic analysis

**Fig. 5:**
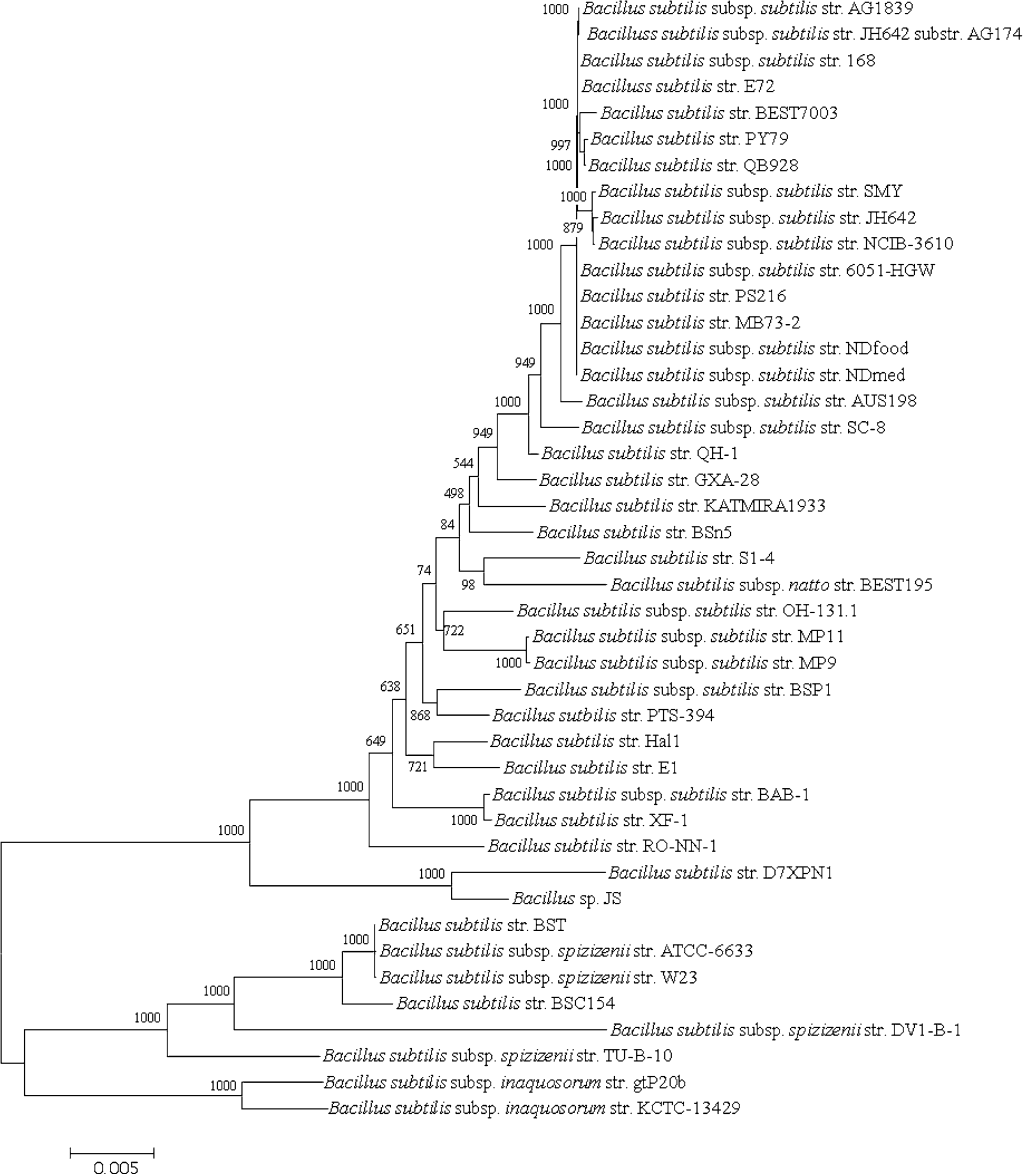
Maximum Likelihood phylogenetic tree of *B. subtilis* (*n* = 43) constructed from a superalignment of sequences from 1724 protein orthologs of *B. subtilis* genomes using the hal pipeline with default parameters. Distances were corrected using the Jone-Taylor (JTT) model with 1000 non-parametric bootstrap replicates. The bootstrap values are represented by numbers at nodes. *Scale bar* indicates 5 differences in every 1000 amino acids (0.5 %). Refer to Table 2 for the type strains in this figure

**Fig. 6:**
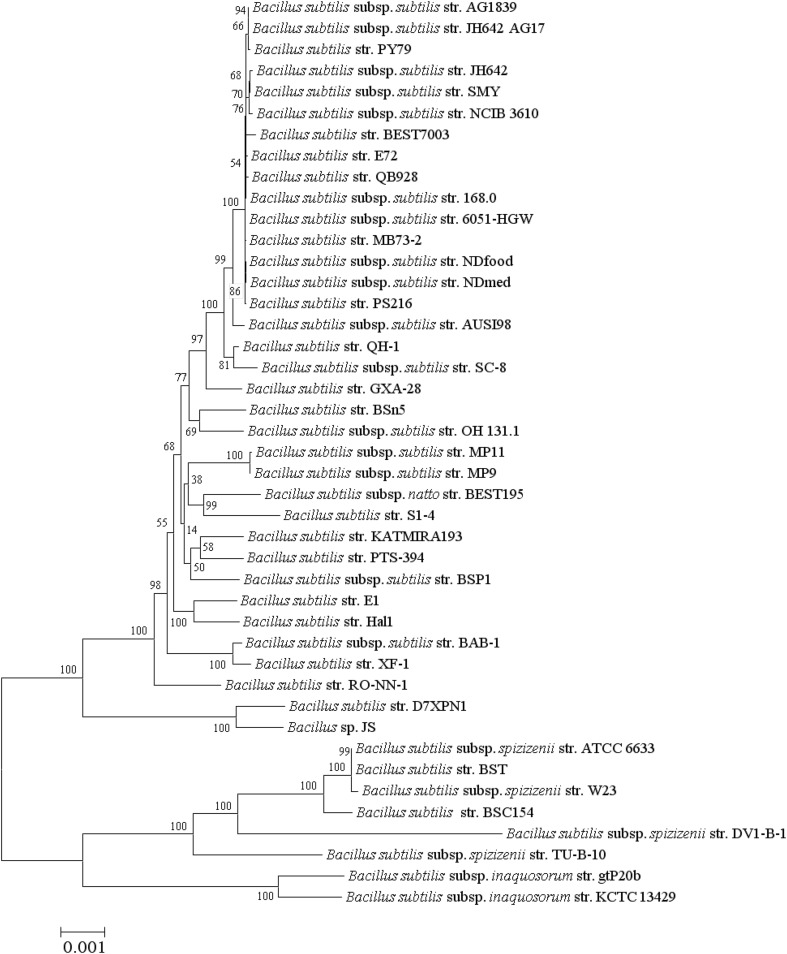
Maximum Likelihood phylogenetic tree of *B. subtilis* (*n* = 43) produced from a superalignment consisting of conserved sequences of 512 protein orthologs (>97 %). Distances were corrected using the Jones -Taylor-Thornton (JTT) model with 1000 non-parametric bootsrap replicates and the values (in percent) are represented at each node. The* scale* *bar* represents a difference of 1 in every 1000 amino acids (0.1 %). Refer to Table 2 for the type strains in this figure

**Fig. 7:**
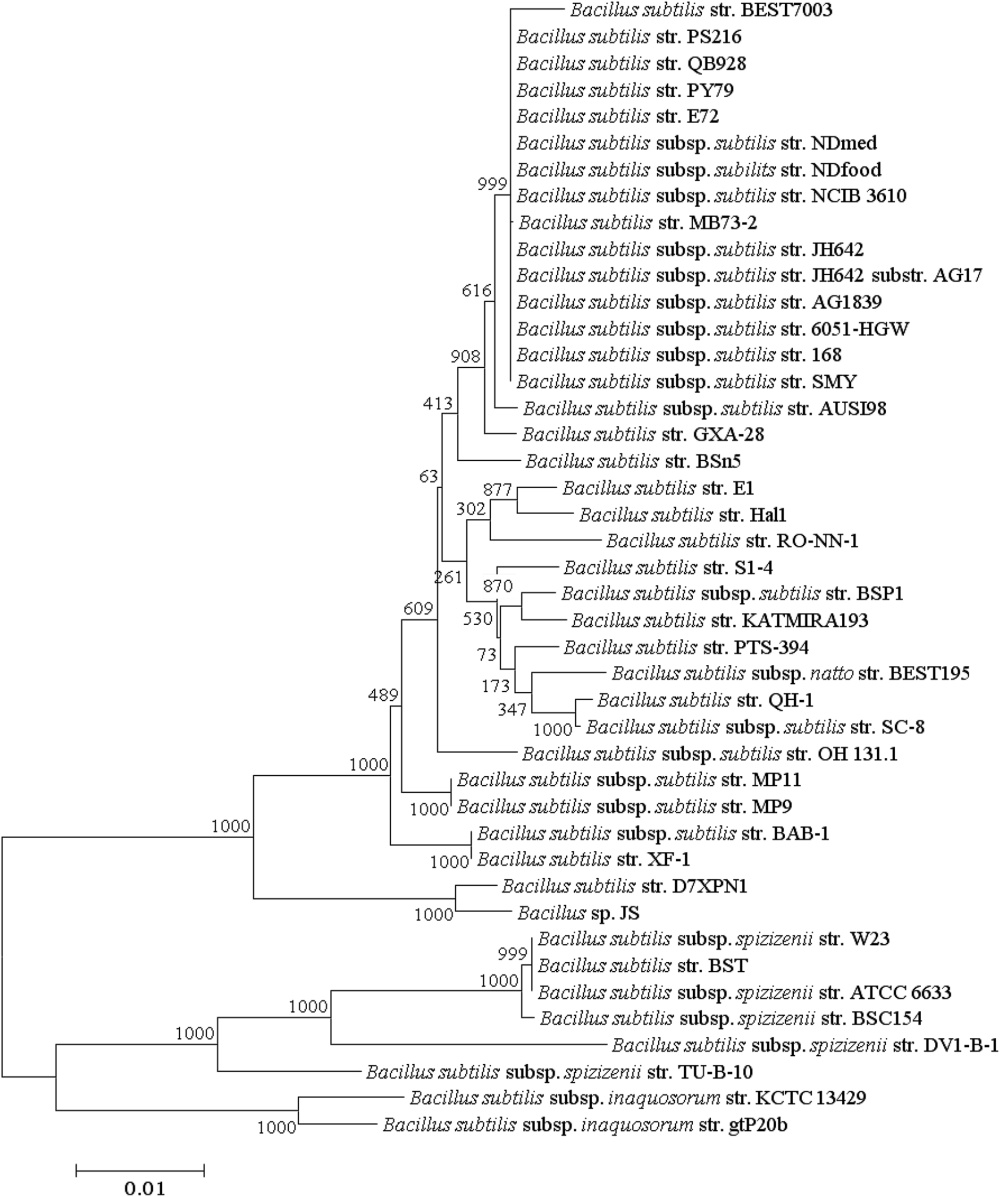
Multi-locus sequence analysis (MLSA) maximum likelihood phylogenetic tree of *B. subtilis* produced from a superalignment of 8861 nucleotide sequences of glpF, pta, purH, pycA, rpoD and tpiA. Distances were corrected using the model of Hasegawa et al. (1985) with 1000 non-parametric bootstrap replicates. The bootstrap values are represented at each node. *Scale bar* represents a difference of 1 in 100 nucleotides (1 %). Refer to Table 2 for the type strains in Table 1

Phylogenomic trees produced from the analysis of 1724 core protein sequence orthologs (436,410 aa) and the more conserved 534 protein sequence orthologs generated from CD-Hit analysis (≥97 % amino acids similarity) are presented in Figs. 5 and 6, respectively and the Multi-Locus Sequence Analysis (MLSA) tree generated from the 6 genes routinely used in Multi-Locus Sequence Typing (MLST) of *B. subtilis* strains is shown in Fig. 7. All 3 trees resolve *Bacillus* strains (*n* = 43) into 4 clusters with the same topology and with 100 % bootstrap values at each branch point of the clusters and the phylogenomic studies supports the results from genome to genome comparisons studies of ANIb and DDH (Fig. 3) though there are slight changes in the topology of the internal branches of cluster 1 representing *B. subtilis* subsp. *subtilis.*

## Discussion

It is well-established that 16S rRNA phylogeny does not readily separate closely related strains of *Bacillus* *subtilis* and including differentiating phenotypic characteristics to phylogenetic data does not necessarily assist in taxonomic delineation. For example, dark pigmented colonies are a distinctive feature of *B. atrophaeus* but some strains of *B. subtilis* also produce such colonies (Nakamura 1989). Several recent studies have discussed the need to establish microbial taxonomy on the basis of information retrieved from microbial genomes especially when 16S rRNA similarity values are >97 % and many computational methods, which can be categorised into two broad groups, have been reported in the literature (Bull et al. 2012; Larsen et al. 2014). Computational methods which rely purely on comparison of nucleotide sequences of genomes, have been recently coined by Chun and Rainey (2014) as Overall Genome Relatedness Indices (OGRI) methods and include ANI, GGDC, GBDP (Genome Blast Distance Phylogeny), Maximal Unique Matches Index (MUMi) whereas methods which rely on comparison of conserved genomic features (e.g. core genes and proteins) where sequence disparities are the result of evolutionary pressures, are known as phylogenomic methods.

In this study we isolated 28 strains from a food-waste degrading commercial bioreactor of which 9 isolates could only be assigned as strains of *B. subtilis* based on 16S rRNA sequence analysis (Fig. 1). We selected strain D7XPN1 as representative of the 9 isolates, sequenced and using OGRI (ANIb and DDH) and phylogenomic methods compared it’s genome with genomes of *B. subtilis* and *B. subtilis*-like strains that had been retrieved from NCBI database. For this we initially downloaded 49 *B.* *subtilis* genomes but were left with only 43 after identifying and removing mixed or misclassified genomes. Of these remaining genomes of 43 *B. subtilis* strains, 21 strains had already been used by Yi et al. (2014) in their studies and our results support their conclusions that ANIb can be used to separate *B. subtilis* into 3 subspecies. They had further suggested that if the threshold ANIb value of 95–96 % were to be used for species delineation than *B. subtilis* subsp. *spizizenii* and *B. subtilis* subsp. *inaquosorum* should be designated as new species. ANIb analysis of the additional 22 strains which were not part of the studies of Yi et al. (2014) revealed that 20 could be assigned to one of the 3 clusters defined by Yi et al. (2014) but a further 2 strains, strains JS and D7XPN1, were closely related to each other (ANib value of 98.8 %) and formed a separate cluster, designated cluster 2. Cluster 2 was a sister branch of cluster 1 and was more closely related to it than to clusters 3 and 4 (ANIb values of 95.6, 93 and 92–94 %, respectively).

Based on the conservative cut off DDH value of 70 %, *B. subtilis* strains (*n* = 43) can be assigned to 4 clusters, each represented by a subspecies and is consistent with the findings of the ANIb analysis. The results also confirm that strains D7XPN1 and JS are related more closely to each other than to members of cluster 1 represented by *B. subtilis* subsp. *subtilis* and supports results from the ANIb analysis. In addition, all the 3 phylogenomic trees generated using 3 different data sets (1724, 534, and 6 core orthologs) resolve *Bacillus* strains (*n* = 43) into 4 clusters with the same topology and with 100 % bootstrap values at each branch point of the clusters and the phylogenomic studies supports the results from genome to genome comparisons studies of ANIb and DDH studies though there are slight changes in the topology of the internal branches of cluster 1 representing *B. subtilis* subsp. *subtilis*.

Phylogenomic studies indicate that there are differences in the gene and protein content of the 43 strains. Doolittle and Zhaxybayeva (2009) have hypothesised that the acquisition of genes can affect changes in an ecological niche and that even the closest relatives could be ecologically distinct ecotypes. Kopac et al. (2014) in their studies on *B. subtilis* subsp. *spizizenii* (cluster 4), which has 6 members, 4 of which were isolated from Death Valley, showed that all the genomes of the four Death Valley strains differed in gene content supporting the hypothesis of Doolittle and Zhaxybayeva (2009) that even the closest relatives could be ecologically distinct ecotypes. However, Kopac et al. (2014) were unable to demonstrate if the acquisition of genes could in fact change the metabolic dynamism of the ecological niche.

It would be interesting to extend the studies to *B. subtilis* subsp. *subtilis* (cluster 1) the most widely represented strains in *B. subtilis* but for the fact that the strains have been isolated from a very wide range of environments and therefore any differences found in the gene content could be considered to be biased due to habitat differences. We have in our study reported here 9 strains of *B. subtilis* isolated from samples taken from the bioreactor on days 0, 1, 7, 17, 21, 29, and 49 of its operation cycle (Table 1), all of which are closely related (16S rRNA similarity >99 % similarity). Strain D7XPN1 reported in our studies, was isolated from a sample collected on day 7 and is a representative of the nine *B. subtilis* strains. Strain D7XPN1 is capable of growing at moderate thermophilic temperatures and contains an array of enzymes for degradation of polysaccharides including a xylanase and an amylase. We intend to sequence and compare the genomes of all the 9 strains and if the hypothesis of Doolittle and Zhaxybayeva (2009) holds true then we should be able to see differences in the gene profiles which would potentially be reflective of the change in environmental conditions in the waste-degrading bioreactor, from day 0 to day 49.

The genome of strain JS isolated from the soil of a pot planted with *Miscanthus* sp. was sequenced and a number of genes associated with plant growth promoting and antifungal activities were identified (Song et al. 2012). In addition, the use of volatile extracts of strain JS were found to reduce the disease in bacterial infected tobacco plants (Kim et al. 2015). Our analysis of the annotations of the genome of strain D7XPN1 has also identified potential plant growth-promoting genes similar to those found in strain JS. The properties of strain D7XPN1 has potential for use as an inoculum source to improve and increase efficiency of the food-waste degradation process at thermophiic temperatures but additionally, it also has the potential for use as a plant growth promoting fertilizer at the completion of the degradation process.

Strains JS and D7XPN1 have not yet been taxonomically validated but based on the OGRI and phylogenomic results reported here, we propose to describe these two strains as members of a newly created subspecies that we designate *B. subtilis* subsp *stecori* of which strain D7XPN1^T^ is the type sub-species. Furthermore, we propose that once more strains of clusters 2, 3 and 4 have been isolated and their genome sequences analysed than if necessary, the reassignment of the *B. subtilis* strains should be reconsidered given the low ANIb (≤95–96 %) and DDH values (<70 %) which demarcate each of the fourclusters.

### Description of *Bacillus subtilis* subsp. *stecoris* subsp. nov

*Bacillus subtilis* subsp. *stecoris* [ste.co.ris.L. gen. n. compost, from which the strain was isolated]. Grows optimally at 45 °C (range 24–50), pH of 7 (range 5–9), and grew in the presence of 7 % NaCl. Facultative anaerobe by fermentation, forms white irregular colonies 1–2 mm in diameter when grown on dTSA, cells are straight rods 4–5 µm length by 1 µm width, and stain Gram positive. Degrades potato starch by amylase activity and expresses partial β-xylanase activity detected when grown on dTSA with x-β-D-xyloside.
